# How Does Thymus Infection by Coxsackievirus Contribute to the Pathogenesis of Type 1 Diabetes?

**DOI:** 10.3389/fimmu.2015.00338

**Published:** 2015-06-30

**Authors:** Hélène Michaux, Henri Martens, Hela Jaïdane, Aymen Halouani, Didier Hober, Vincent Geenen

**Affiliations:** ^1^Department of Biomedical and Preclinical Sciences, GIGA-I^ 3^ Center of Immunoendocrinology, GIGA Research Institute, University of Liege, Liege, Belgium; ^2^Laboratory of Virology LR99ES27, School of Pharmacy, University of Monastir, Monastir, Tunisia; ^3^Faculty of Sciences of Tunis, University of Tunis El Manar, Tunis, Tunisia; ^4^Laboratory of Virology EA3610, Centre Hospitalier Régional Universitaire de Lille, University of Lille 2, Lille, France

**Keywords:** enterovirus, coxsackievirus, thymus, self-tolerance, type 1 diabetes, insulin family, insulin-like growth factor 2

## Abstract

Through synthesis and presentation of neuroendocrine self-antigens by major histocompatibility complex proteins, thymic epithelial cells (TECs) play a crucial role in programing central immune self-tolerance to neuroendocrine functions. Insulin-like growth factor-2 (IGF-2) is the dominant gene/polypeptide of the insulin family that is expressed in TECs from different animal species and humans. *Igf2* transcription is defective in the thymus of diabetes-prone bio-breeding rats, and tolerance to insulin is severely decreased in *Igf2*^−/−^ mice. For more than 15 years now, our group is investigating the hypothesis that, besides a pancreotropic action, infection by coxsackievirus B4 (CV-B4) could implicate the thymus as well, and interfere with the intrathymic programing of central tolerance to the insulin family and secondarily to insulin-secreting islet β cells. In this perspective, we have demonstrated that a productive infection of the thymus occurs after oral CV-B4 inoculation of mice. Moreover, our most recent data have demonstrated that CV-B4 infection of a murine medullary (m) TEC line induces a significant decrease in *Igf2* expression and IGF-2 production. In these conditions, *Igf1* expression was much less affected by CV-B4 infection, while *Ins2* transcription was not detected in this cell line. Through the inhibition of *Igf2* expression in TECs, CV-B4 infection could lead to a breakdown of central immune tolerance to the insulin family and promote an autoimmune response against insulin-secreting islet β cells. Our major research objective now is to understand the molecular mechanisms by which CV-B4 infection of TECs leads to a major decrease in *Igf2* expression in these cells.

## Introduction

The major genetic determinants of type 1 diabetes (T1D) are the class II major histocompatibility complex (MHC) on chromosome 6 – which accounts for almost 50% of the genetic susceptibility – as well as a number of non-MHC genes, including the variable number of tandem repeat (VNTR) alleles upstream of the *INS/IGF2* (*IDDM2*) locus, *PTPN22*, *CCR5*, *IL2RA*, *IL10*, and *CTLA4*. However, only 10% of the individuals bearing a genetic predisposition will develop T1D, and more than 50% of monozygotic twins are discordant for the disease, which illustrates the implication of environmental influences in T1D pathogenesis (1) as for all autoimmune diseases.

Type 1 diabetes occurrence has been related to a number of viruses but epidemiological studies have provided the strongest evidence that enteroviral infections, in particular, by coxsackievirus B (CV-B), are frequent events preceding T1D onset (2–7). Human enteroviruses include human pathogens, such as poliovirus, CV-B, rhinovirus, and echovirus. Using RT-PCR detection, CV-B genome was detected in 5 out of 12 (42%) newly diagnosed T1D patients and in 1 of 12 (8%) patients during the course of T1D. None of T2D patients and none of 15 healthy controls had enterovirus sequences in their blood (8). CV-B4 E2 can persistently infect human β cells (9) and a CV-B4 variant infects β cells leading to a disturbance of proinsulin synthesis and insulin secretion (10). The mechanism most accredited to explain the link between CV-B infection and T1D is a specific tropism of the virus for insulin-secreting islet β cells (11) – that is, mediated by their expression of the specific virus receptor – and a bystander activation of autoreactive T cells by antigens released by β cells after their damage caused by CV-B infection (12). Another crucial study has shown that CV-B4 is able to infect β cells in patients with T1D and that such infection is associated with both inflammation and severe β-cell functional disturbance (13). The persistent aspect of enterovirus infection is also an important factor to take into account [for a complete review, see Ref. (14)]. Very recently, this scenario received a strong support through the Diabetes Virus Detection (DiViD) study that detected a low-grade enteroviral infection in the islets of Langerhans collected from living patients newly diagnosed with T1D (15). This study does not prove a causal relationship between enterovirus infection and T1D, but is the first to detect enterovirus in pancreatic islets from patients close to the time of their diagnosis of T1D. The association between T1D and viral infections has also been previously reinforced by a genetic linkage between T1D susceptibility and host determinants of the antiviral response, such as the antiviral oligoadenylate synthase (OAS1) and the interferon-induced helicase (IFIH1), which intervene in innate immunity by recognition of RNA genome of picornaviruses, such as enteroviruses (16, 17). Besides this pancreotropism of CV-B, we have been exploring for a long time another mechanism that could play an essential and complimentary role in the development of the diabetogenic autoimmune response, namely, thymus infection.

## Thymus-Dependent Central Self-Tolerance to Islet β Cells

As previously demonstrated that the thymus epithelium plays a unique role in programing central self-tolerance to neuroendocrine functions [complete reviews in Ref. (18–20)], as well as to many tissue-related antigens (21). Following gene transcription in the thymus, neuroendocrine precursors are processed not according to the classical model of neurosecretion but for presentation by, or in association with, the thymic MHC machinery. In the thymus, MHC presentation of neuroendocrine self-peptides promotes two intimately associated but paradoxical events: (1) negative selection and deletion of self-reactive T cell clones and (2) Generation of self-specific regulatory T (tTreg) cells that are able to inhibit in the periphery those “forbidden” self-reactive T cells that escaped thymic clonal deletion. The AutoImmune REgulator (AIRE) protein controls intrathymic transcription of neuroendocrine genes, including all the members of the insulin gene family (22) that are transcribed in the murine thymus according to the following hierarchy: *Igf2* > *Igf1* > *Ins2* > *Ins1*. Thymic self-antigen expression and AIRE function are also regulated by epigenetic and post-translational mechanisms (23).

There is now mounting evidence that a defect in intrathymic negative selection is implicated in the development of autoimmune endocrine diseases, such as T1D (24–27), although this is still discussed for the non-obese diabetic (NOD) thymus (28, 29). Contrary to *Igf1* and *Ins2*, *Igf2* transcription is defective in the thymus of diabetes-prone of bio-breeding (BB) rats (30), one of the two animal models of T1D with the NOD mouse. In humans, *INS* transcripts are measured at a lower level in the thymus from fetuses with short class I VNTR alleles, the second genetic trait (*IDDM2*) of T1D susceptibility (31, 32). Both VNTR alleles and AIRE determine the concentration of *INS* transcripts in the human thymus (33). In the mouse, *Ins2* is predominantly transcribed in the thymus, while *Ins1* expression is dominant in islet β cells, which leads to a higher immunological tolerance to *Ins2*. This explains why the breeding of *Ins2*^−/−^ mice onto the NOD background accelerates insulitis and diabetes onset (34), whereas insulitis and diabetes are markedly inhibited in *Ins1*^−/−^ congenic NOD mice (35). There is now firm evidence that *Ins1* codes for the primary insulin-derived autoantigenic epitopes tackled by the autoimmune diabetogenic process (36, 37). In addition, there is a very rapid onset of autoimmune diabetes after a thymus-specific *Ins1* and *Ins2* deletion resulting from the crossing of *Ins1*^−/−^ mice with mice presenting a specific *Ins2* deletion in *Aire*-expressing medullary thymic epithelial cells (TECs) (38). The insulin transactivator *Mafa* also regulates *Ins2* transcription in the thymus and targeted *Mafa* disruption induces appearance of anti-islet antibodies (39).

## Tolerogenic Properties of IGF-2: Multiple Facets

Given the direct relationship between the expression level of a protein/peptide in the thymus and the immunological tolerance to this protein/peptide (40), the hierarchical profile of the intrathymic expression of insulin-related peptides (IGF-2 > IGF-1 > insulin) suggests that tolerance to insulin-like growth factor-2 (IGF-2) is high and that tolerance to insulin is low. This is indirectly supported by the fact that insulin is the primary autoantigen of T1D (36, 37) while no autoimmune response against IGF-2 has ever been reported. Conversely, the highly immunogenic properties of insulin might actually be related to its very low expression in rare medullary (m) TEC subsets. Recently, the alternate variant INS–IGF-2 has been identified as a novel autoantigen in T1D (41), but there is still no data about the expression of this hybrid protein in thymic epithelium. Spontaneous autoimmune diabetes does not develop in *Igf2*^−/−^ mice although these mice display a marked lower tolerance to insulin, which evidences that *Igf2* expression mediates cross-tolerance to insulin and is required for the programing of a complete immunological tolerance to this protein (42). The homologous sequences Ins B9-23 and IGF-2 B11-25 compete for binding to the MHC-II DQ8 allele, and their presentation to PBMCs isolated from DQ8^+^ T1D adolescents induce distinct cytokine profiles with a regulatory profile for IGF-2 B11-25 that is not observed for Ins B9-23 (43). Two recent studies have further evidenced the tolerogenic properties of IGF-2 by enhancement of Treg cell functions in an experimental model of food allergy (44), as well as promotion of antigen-specific Breg cell properties (45).

Our studies have also shown that the blockage of IGF-mediated signaling in the thymus severely interferes with T-cell growth and differentiation blocks T-cell differentiation (46), which was further confirmed by the demonstration that an antibody to CD222 (the IGF-2 receptor, an endosomal transporter that regulates protein trafficking) plays a central function in the initiation of T-cell signal transduction (47).

Therefore, the predominant expression of IGF-2 in the thymus is not only associated with a higher immunological tolerance to this protein but also seems to confer significant tolerogenic properties to IGF-2- and IGF-2-derived antigen sequences. On these experimental bases, we have proposed the novel concept of “negative self-vaccination” that is under current development through DNA vaccine methodology (48).

## Thymus Infection by Enteroviruses

Given the programing of self-tolerance to islet β cells in the thymus and its defect in the development of the autoimmune diabetogenic response, we investigated the question of a putative role played by an enteroviral infection in an acquired dysfunction of the three major properties of this primary lymphoid organ: thymopoiesis, establishment of central self-tolerance, and generation of self-antigen-specific tTreg cells. A persistent replication of CV-B4 E2 (a “diabetogenic” CV-B strain) and JBV (a prototype CV-B strain) in primary cultures of human TECs was demonstrated by detection of positive- and negative-strand viral RNA in extracts from cell cultures, by immunofluorescence staining of the VP1 capsid protein, and by release of infectious particles up to 30 days after culture inoculation without any apparent cytolytic effect. The persistence of CV-B4 infection was associated with an increased rate of TEC proliferation and with an increase in the secretion of the cytokines IL-6, LIF, and GM-CSF in the supernatants. CV-B4 replication was not restricted to the CV-B4 E2 strain and did not depend on the genetic background of the host. However, cytokine secretion in human TEC cultures infected with CV-B4 E2 was higher than in cultures infected with CV-B4 JBV (49). Therefore, although they are considered as cytolytic viruses, enteroviruses can infect persistently some tissues, such as thymus and pancreas.

Coxsackievirus B4 E2 is also able to infect human fetal thymic organ cultures (FTOC). Viral RNA was detected by quantitative RT-PCR in CV-B4 E2-infected human FTOC, which supported high yields of virus production, as well as in flow-sorted thymic T cell populations for 7 days after infection. In FTOC, double positive CD4^+^CD8^+^ thymocytes were the principal target cells of infection and were progressively and severely depleted with no sign of apoptosis. Of note, massive thymic depletion of developing T cells and the subsequent CD4^+^CD25^+^ tTreg cells was shown previously to result in systemic autoimmunity (50). CV-B4 E2 replication caused a major up-regulation of MHC class I expression on thymic T cells and TECs. This MHC class I up-regulation was correlated with markers of CV-B4 infection (viral RNA quantification, release of infectious particles), and this was the result of a direct infection rather than caused by production of soluble factors, such as interferon-α (51). Interestingly, Krogvold et al. also reported an overexpression of MHC class I in the islets of all the patients included in their recent study (15). CV-B4 E2 was similarly shown to disturb T-cell differentiation in infected murine FTOC (52). In concordance with previous observations (53), CV-B4 oral inoculation of outbred mice results in a systemic spreading of viral RNA and a detection of viral RNA in thymus, spleen and blood up to 70 days after inoculation (54). Finally, CV-B4 infection of a murine mTEC line induces a dramatic decrease in *Igf2* transcription and IGF-2 production in long-term cultures of this cell line, while *Igf1* transcripts were much less affected and *Ins2* transcripts were not detected in these experimental conditions (55). Inoculation of the mTEC line with CV-B3, CV-B4 JVB, or echovirus 1 also induced a decrease in IGF-2 production, while herpes simplex virus 1 stimulated IGF-2 production. As already cited, a defect of *Igf2* expression in the thymus was suggested to play a role in the development of autoimmune diabetes in the diabetes-prone BB rat (30). Although these effects need to be reproduced *in vivo*, they strongly support our hypothesis that CV-B4 infection of the thymus could disrupt central self-tolerance to the insulin family, and could also enhance CV-B4 virulence through induction of central immunological tolerance to this virus. We are currently investigating the molecular mechanisms responsible for the CV-B-induced decrease of thymic IGF-2 expression in this mTEC line and *in vivo* after oral inoculation of CD1 mice. Since the CV-B-mediated effects in mTEC line are more pronounced on IGF-2 protein than on *Igf2* transcription, we concluded that post-transcriptional and/or post-translational mechanisms could be both involved.

As previously discussed by Zinkernagel (56), fetal exposure to maternal enterovirus infections should also be taken into account. One study has shown that enterovirus infection during the first trimester of pregnancy is not associated with a higher risk for T1D in the childhood (57), but another one has evidenced that such maternal enterovirus infection was a risk factor in offspring diagnosed with T1D between 15 and 30 years of age (58). More recently, a study has investigated that the effects of CV-B4 E2 oral inoculation of CD1 mice at days 4, 10, or 17 of gestation. Severe inflammation of the pancreas and higher glucose blood levels were observed only when dams were previously infected and, in particular, at day 17, thus, in the late phase of pregnancy (59). CV-B4 E2 oral inoculation of pregnant mice is also associated with fetal thymus infection and disturbance of T-cell differentiation (Jaïdane, personal communication). Obviously, the question of materno-fetal transmission of enterovirus infection highly deserves to be further investigated.

## Conclusion: A Model Associating CV-B-Induced Dysfunction of Central Tolerance and Peripheral Bystander Activation

**Figure 1:**
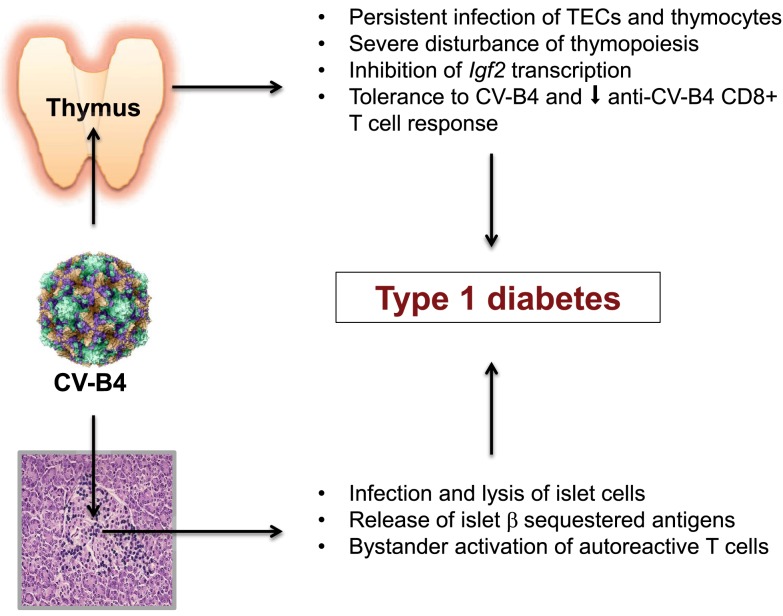
**Coxsackievirus B4 persistent infection of thymus and pancreatic islets are closely associated and implicated in T1D pathogenesis**.

In addition to the necessity of standardization for the serological and RT-PCR detection of CV-B infection as recommended by Gale and Atkinson (60), there is also an urgent need for a thorough investigation of the relationships between CV-B and the host immune system (Figure 1). What is our current knowledge about this point? CV-B4 is able to persistently infect α and β cells in human pancreatic islets, and to cause functional impairment and β-cell death characterized by nuclear pyknosis. The CV-B4-induced damage to the islet cells causes release and presentation of sequestered islet antigens. Through bystander activation, autoreactive T cells initiate the diabetogenic autoimmune process. Now, with regard to the origin of these autoreactive T cells, more and more experimental evidence points to the generation in the thymus of “forbidden” T cell clones due to a failure of the central tolerogenic mechanisms. This thymus defect results in a progressive enrichment of the peripheral T cell repertoire with self-reactive T cells and a decreased generation of self-antigen tTreg cells. From our collaborative work, it appears that CV-B4 is also able to persistently infect the epithelial and lymphoid compartments of the thymus. CV-B4 infection of the thymus leads to increased secretion of diverse cytokines synthesized in TECs, to a severe depletion of double positive CD4^+^CD8^+^ thymocytes, and to marked up-regulation of MHC class I molecules expressed by TECs and double positive thymic T cells. Moreover, CV-B4 infection of a murine mTEC line induces a marked decrease in *Igf2* transcription and IGF-2 production. Therefore, a CV-B4 persistent infection of the thymus may lead to significant thymus and immune dysregulation that associates:

- A significant impairment of thymus-dependent self-tolerance issued from the decrease in the presentation of insulin family related self-antigens, and putatively a direct viral interference with self-antigen presentation (61).
- An induction of central tolerance to CV-B4 and a secondary decrease of anti-CV-B4 CD8^+^ T-cell mediated response, so that further exposure to the virus could promote more severe damage to the peripheral target tissues.

If further research confirmed such rational assumption based on our new knowledge of thymus functions, then an anti-CV-B4 vaccination could be considered as a strategy for T1D prevention in regions with a high incidence of this disease such as in Scandinavian countries (62).

## Conflict of Interest Statement

The authors declare that the research was conducted in the absence of any commercial or financial relationships that could be construed as a potential conflict of interest.

## References

[1] Steck AK Rewers MJ Genetics of type 1 diabetes. Clin Chem (2011) 57:176–85. 10.1373/clinchem.2010.148221 21205883 PMC4874193

[2] Hyöti H Hiltunen M Knip M Laakonen M Vähäsalo P Karjalainen J A prospective role of coxsackie B and enterovirus infections in the pathogenesis of IDDM. Childhood Diabetes in Finland (DiMe) Study Group. Diabetes (1995) 44:652–67. 10.2337/diabetes.44.6.652 7789630

[3] Yeung WC Rawlinson WD Craig ME. Enterovirus infection and type 1 diabetes mellitus: systematic review and meta-analysis of observational molecular studies. BMJ (2011) 342:d35. 10.1136/bmj.d35 21292721 PMC3033438

[4] Oikarinen S Tauriainen S Hober D Lucas B Vazeou A Sioofy-Khojine A VirDiab Study Group. Virus antibody survey in different European populations indicates risk association between coxsackievirus B1 and type 1 diabetes. Diabetes (2014) 63:655–62. 10.2337/db13-0620 24009257

[5] Jaïdane H Hober D. Role of coxsackievirus B4 in the pathogenesis of type 1 diabetes. Diabetes Metab (2008) 34:537–48. 10.1016/j.diabet.2008.05.008 18951821

[6] Hober D Sauter P. Pathogenesis of type 1 diabetes mellitus: interplay between enterovirus and host. Nat Rev Endocrinol (2010) 6:279–89. 10.1038/nrendo.2010.27 20351698

[7] Jaïdane H Sauter P Sane F Goffard A Gharbi J Hober D. Enteroviruses and type 1 diabetes: towards a better understanding of the relationship. Rev Med Virol (2010) 20:265–80. 10.1002/rmv.647 20629044

[8] Chehadeh W Weill J Vantyghem MC Alm G Lefebvre J Wattré P Increased level of interferon-alpha in blood of patients with insulin-dependent diabetes mellitus: relationship with coxsackie B virus infection. J Infect Dis (2000) 181:1929–39. 10.1086/315516 10837172

[9] Chehadeh W Kerr-Conte J Patou F Alm G Lefebvre J Wattré P Persistent infection of human pancreatic islets by coxsackievirus B is associated with alpha-interferon synthesis in beta cells. J Virol (2000) 74:10153–64. 10.1128/JVI.74.21.10153-10164.2000 11024144 PMC102054

[10] Yin H Berg AK Westman J Hellerström C Frisk G Complete nucleotide sequence of a coxsackievirus B4 strain capable of establishing persistent infection in human pancreatic islets: effects on insulin release, proinsulin synthesis, and cell morphology. J Med Virol (2002) 68:544–7. 10.1002/jmv.10236 12376963

[11] Roivanen M Rasilainen S Ylipaasto P Nissinen R Ustinov J Bouwens L Mechanisms of coxsackievirus-induced damage to human pancreatic beta-cells. J Clin Endocrinol Metab (2000) 85:432–40. 10.1210/jc.85.1.432 10634421

[12] Horwitz MS Ilic A Fine C Rodriguez E Sarvetnick N. Presented antigen from damaged pancreatic beta cells activates autoreactive T cells in virus-mediated autoimmune diabetes. J Clin Invest (2002) 109:79–87. 10.1172/JCI0211198 11781353 PMC150813

[13] Dotta F Censini S van Halteren AGS Marselli L Masini M Dionisi S Coxsackie B4 virus infection of β cells and natural killer cell insulitis in recent-onset type 1 diabetic patients. Proc Natl Acad Sci U S A (2007) 104:5115–20. 10.1073/pnas.0700442104 17360338 PMC1829272

[14] Alidjinou EK Sané F Engelmann I Geenen V Hober D. Enterovrus persistence as a mechanism in the pathogenesis of type 1 diabetes. Discov Med (2014) 18:273–82. 25425468

[15] Krogvold L Bjorn E Buanes T Frisk G Skog O Anagandula M Detection of a low-grade enteroviral infection in the islets of Langerhans of living patients newly diagnosed with type 1 diabetes. Diabetes (2015) 64:1682–7. 10.2337/db14-1370 25422108

[16] Field LL Bonnevie-Nielsen V Pociot F Lu S Nielsen TB Beck-Nielsen H. *OAS1* splice site polymorphism controlling antiviral enzyme activity influences susceptibility to type 1 diabetes. Diabetes (2005) 54:1588–91. 10.2337/diabetes.54.5.1588 15855350

[17] Smyth DJ Cooper JD Bailey R Field S Burren O Smink LJ A genome-vide association study of nonsynonymous SNPs identifies a type 1 diabetes locus in the interferon-induced helicase (IFIH1) region. Nat Genet (2006) 38:617–9. 10.1038/ng1800 16699517

[18] Bonomo A Matzinger P. Thymus epithelium induces tissue-specific tolerance. J Exp Med (1993) 177:1153–64. 10.1084/jem.177.4.1153 8459209 PMC2190970

[19] Martens H Goxe B Geenen V The thymic repertoire of neuroendocrine self-antigens: physiological implications in T-cell life and death. Immunol Today (1996) 17:312–7. 10.1016/0167-5699(96)10023-2 8763816

[20] Geenen V Bodart G Henry S Michaux H Dardenne O Charlet-Renard C Programming of neuroendocrine self in the thymus and its defect in the development of neuroendocrine autoimmunity. Front Neurosci (2013) 7:187. 10.3389/fnins.2013.00187 24137108 PMC3797387

[21] Kyewski B Klein L The central role of central tolerance. Annu Rev Immunol (2006) 24:571–605. 10.1146/annurev.immunol.23.021704.115601 16551260

[22] Anderson MS Venanzi ES Klein L Chen Z Berzin SP Turley SJ Projection of an immunological self shadow in the thymus by the Aire protein. Science (2002) 298:1395–401. 10.1126/science.1075958 12376594

[23] Ucar O Rattay K. Promiscuous gene expression in the thymus: a matter of epigenetics, miRNA and more? Front Immunol (2015) 6:93. 10.3389/fimmu.2015.00093 25784915 PMC4347492

[24] Like AA Kislaukis E Williams RM Rossini AA. Neonatal thymectomy prevents spontaneous diabetes in the BB:W rat. Science (1982) 216:644–6. 10.1126/science.7041259 7041259

[25] Georgiou HM Bellgrau D. Induction of insulitis in athymic (nude) mice. The effect of NOD thymus and pancreas transplantation. Diabetes (1995) 44:49–59. 10.2337/diabetes.44.1.49 7813814

[26] Thomas-Vaslin V Damotte D Coltey M Le Douarin NM Coutinho A Salaün J. Abnormal T cell selection on nod thymic epithelium is sufficient to induce autoimmune manifestations in C57BL/6 athymic nude mice. Proc Natl Acad Sci U S A (1997) 94:4598–603. 10.1073/pnas.94.9.4598 9114036 PMC20769

[27] Kishimoto H Sprent J. A defect in central tolerance in NOD mice. Nat Immunol (2001) 2:1025–31. 10.1038/ni726 11668341

[28] Zucchelli S Holler P Yamagata T Roy M Benoist C Mathis D. Defective central tolerance induction in NOD mice: genomics and genetics. Immunity (2005) 22:385–96. 10.1016/j.immuni.2005.01.015 15780994

[29] Mingueneau M Jiand W Feuerer M Mathis D Benoist C. Thymic negative selection is functional in NOD mice. J Exp Med (2012) 209:623–37. 10.1084/jem.20112593 22329992 PMC3302233

[30] Kecha-Kamoun O Achour I Martens H Collette J Lefebvre PJ Greiner DL Thymic expression of insulin-related genes in an animal of type 1 diabetes. Diabetes Metab Res Rev (2001) 17:146–52. 10.1002/dmmr.182 11307180

[31] Vafiadis P Bennett ST Todd JA Nadeau J Grabs R Goodyer CG Insulin expression in human thymus is modulated by *INS* VNTR alleles at the *IDDM2* locus. Nat Genet (1997) 15:289–92. 10.1038/ng0397-289 9054944

[32] Pugliese A Zeller M Fernandez A Jr Zalcberg LJ Bartlett RJ Ricordi C The insulin gene is transcribed in the human thymus and transcription levels correlate with allelic variation at the *INS* VNTR-*IDDM2* susceptibility locus for type 1 diabetes. Nat Genet (1997) 15:293–7. 10.1038/ng0397-293 9054945

[33] Sabater L Ferrer-Francesch X Sospedra M Caro P Juan M Pujol-Borrell R. Insulin alleles and autoimmune regulator (AIRE) gene both influence insulin expression in the thymus. J Autoimmun (2005) 25:312–8. 10.1016/j.aut.2005.08.006 16246524

[34] Thebault-Baumont K Dubois-Laforgue D Krief P Briand JP Halbout P Vallon-Geoffroy K Acceleration of type 1 diabetes mellitus in proinsulin 2-deficient mice. J Clin Invest (2003) 111:851–7. 10.1172/JCI16584 12639991 PMC153768

[35] Moriyama H Abiru N Paronen J Sikora K Liu E Miao D Evidence for a primary islet autoantigen (proinsulin 1) for insulitis and diabetes in the nonobese diabetic mice. Proc Natl Acad Sci U S A (2003) 100:10376–81. 10.1073/pnas.1834450100 12925730 PMC193569

[36] Nakayama M Abiru N Moriyama H Babaya N Liu E Miao D Prime role for an insulin epitope in the development of type 1 diabetes in NOD mice. Nature (2005) 435:220–3. 10.1038/nature03523 15889095 PMC1364531

[37] Kent SC Chen Y Bregoli L Clemmings SM Kenyon NS Ricordi C Expanded T cells from pancreatic lymph nodes of type 1 diabetic subjects recognize an insulin epitope. Nature (2005) 435:224–8. 10.1038/nature03625 15889096

[38] Fan Y Rudert WA Grupillo M He J Sisino G Trucco M. Thymus-specific deletion of insulin induces autoimmune diabetes. EMBO J (2009) 28:2812–24. 10.1038/emboj.2009.212 19680229 PMC2750011

[39] Noso S Kataoka K Kawabata Y Babaya N Hiromine Y Yamaji K Insulin transactivator MafA regulates intrathymic transcription of insulin and affects susceptibility to type 1 diabetes. Diabetes (2010) 59:2579–87. 10.2337/db10-0476 20682694 PMC3279543

[40] Ashton-Rickardt P Bandeira A Delaney JR Van Kaer L Pircher HP Zinkernagel RM Evidence for a differential avidity model of T cell selection in the thymus. Cell (1994) 76:651–63. 10.1016/0092-8674(94)90505-3 8124708

[41] Kanatsuma N Taneera J Vaziri-Sani F Wierup N Larsson HE Delli A Autoimmunity against INS-IGF2 protein expressed in human pancreatic islets. J Biol Chem (2013) 288:29013–23. 10.1074/jbc.M113.478222 23935095 PMC3789998

[42] Hansenne I Charlet-Renard C Greimers R Geenen V. Dendritic cell differentiation and tolerance to insulin-related peptides in *Igf2*-deficient mice. J Immunol (2006) 176:4651–7. 10.4049/jimmunol.176.8.4651 16585557

[43] Geenen V Louis C Martens H The Belgian diabetes registry. An insulin-like growth factor 2-derived self-antigen inducing a regulatory cytokine profile to peripheral blood mononuclear cells from DQ8+ type 1 diabetic adolescents: preliminary design of a thymus-based tolerogenic self-vaccination. Ann N Y Acad Sci (2004) 1037:59–64. 10.1196/annals.1337.008 15699493

[44] Yang G Geng XR Song JP Wu Y Yan H Zhan Z Insulin-like growth factor 2 enhances regulatory T-cell functions and suppresses food allergy in an experimental model. J Allergy ClinImmunol (2014) 133:1702–8. 10.1016/j.jaci.2014.02.019 24698315

[45] Geng XR Yang G Li M Song JP Liu ZQ Qiu S Insulin-like growth factor 2 enhances functions of antigen (Ag)-specific regulatory B cells. J Biol Chem (2014) 289:17941–50. 10.1074/jbc.M113.515262 24811165 PMC4067224

[46] Kecha O Brilot F Martens H Franchimont N Renard C Greimers R Involvement of insulin-like growth factors in early T cell development: a study using fetal thymic organ cultures. Endocrinology (2000) 141:1209–17. 10.210/en.141.3.1209 10698198

[47] Pfister K Forster F Paster W Supper V Ohradanova-Repic A Eckerstorfer P The late endosomal transporter CD222 directs the spatial distribution and activity of Lck. J Immunol (2014) 193:2718–32. 10.4049/jimmunol.1303349 25127865

[48] Geenen V Mottet M Dardenne O Kermani H Martens H Francois JM Thymic self-antigens for the design of a negative/tolerogenic self-vaccination against type 1 diabetes. Curr Opin Pharmacol (2010) 10:461–72. 10.1016/j.coph.2010.04.005 20434402

[49] Brilot F Chehadeh W Charlet-Renard C Martens H Geenen V Hober D. Persistent infection of human thymic epithelial cells by coxsackievirus B4. J Virol (2002) 76:5260–5. 10.1128/JVI.76.10.5260-5265.2002 11967339 PMC136150

[50] Shih FF Mandik-Nayak L Wipke BT Allen PM. Massive thymic deletion results in systemic autoimmunity through elimination of CD4+CD25+ T regulatory cells. J Exp Med (2004) 199:323–35. 10.1084/jem.20031137 14744995 PMC2211803

[51] Brilot F Geenen V Hober D Stoddart C. Coxsackievirus B4 infection of human fetal thymus cells. J Virol (2004) 78:9854–61. 10.1128/JVI.78.18.9854-9861.2004 15331720 PMC514990

[52] Brilot F Jaïdane H Geenen V Hober D. Coxsackievirus B4 infection of murine fetal thymus organ cultures. J Med Virol (2008) 80:659–66. 10.1002/jmv.21016 18297721

[53] Chatterjee NK Hou J Dockstader P Charbonneau T. Coxsackievirus B4 infection alters thymic, splenic, and peripheral lymphocyte repertoire preceding onset of hyperglycemia in mice. J Med Virol (1992) 38:124–31. 10.1002/jmv.1890380210 1334127

[54] Jaïdane H Gharbi J Lobert PE Lucas B Hiar R M’Hadheb MB Prolonged viral RNA detection in blood and lymphoid tissues from coxsackievirus B4-E2 orally-inoculated mice. Microbiol Immunol (2006) 50:971–4. 10.1111/j.1348-0421.2006.tb03874.x 17179665

[55] Jaïdane H Caloone D Lobert PE Sané F Dardenne O Naquet P Persistent infection of thymic epithelial cells with coxsackievirus B4 results in decreased expression of type 2 insulin-like growth factor. J Virol (2012) 86:11151–62. 10.1128/JVI.00726-12 22855493 PMC3457166

[56] Zinkernagel RM Maternal antibodies, childhood infections, and autoimmune diseases. N Engl J Med (2001) 345:1331–5. 10.1056/NEJMra012493 11794153

[57] Viskari HR Roivainen M Reunanen A Pitkäniemi J Sadeharju K Koskela P Maternal first-trimester enterovirus infection and future risk of type 1 diabetes in the exposed fetus. Diabetes (2002) 51:2568–71. 10.2337/diabetes.51.8.2568 12145172

[58] Elfving M Svensson J Oikarinen S Jonsson B Olofsson P Sundkvist G Maternal enterovirus infection during pregnancy as a risk factor in offspring diagnosed with type 1 diabetes between 15 and 30 years of age. Exp Diabetes Res (2008) 2008:271958. 10.1155/2008/271958 18670622 PMC2491699

[59] Bopegamage S Precechtelova J Marosova L Stipalova D Sojka M Borsanyiova M Outcome of challenge with coxsackievirus B4 in young mice after maternal infection with the same virus during gestation. FEMS Immunol Med Microbiol (2012) 64:184–90. 10.1111/j.1574-695X.2011.00886.x 22066931

[60] Gale EAM Atkinson M A piece of nucleic acid surrounded by controversy: coxsackievirus and the cause of type 1 diabetes. Diabet Med (2004) 21:503–6. 10.1111/j.1464-5491.2004.01181.x 15154931

[61] Yewdell JY Hill AB Viral interference with antigen presentation. Nat Immunol (2002) 3:1019–25. 10.1038/ni1102-1019 12407410

[62] Karvonen M Viik-Kajander M Moltchanova E Libman I LaPorte R Tuomilehto J. Incidence of childhood type 1 diabetes worldwide. Diabetes Mondiale (DiaMond) Project Group. Diabetes Care (2000) 23:1516–26. 10.2337/diacare.23.10.1516 11023146

